# Oleocanthal Attenuates Metastatic Castration-Resistant Prostate Cancer Progression and Recurrence by Targeting SMYD2

**DOI:** 10.3390/cancers14143542

**Published:** 2022-07-21

**Authors:** Abu Bakar Siddique, Hassan Y. Ebrahim, Afsana Tajmim, Judy Ann King, Khaldoun S. Abdelwahed, Zakaria Y. Abd Elmageed, Khalid A. El Sayed

**Affiliations:** 1 School of Basic Pharmaceutical and Toxicological Sciences, College of Pharmacy, University of Louisiana Monroe, 1800 Bienville Drive, Monroe, LA 71201, USA; siddique@ulm.edu (A.B.S.); ebrahim@ulm.edu (H.Y.E.); tajmima@warhawks.ulm.edu (A.T.); abdelwks@warhawks.ulm.edu (K.S.A.); 2 Department of Pathology and Translational Pathobiology, LSU Health Shreveport, 1501 Kings Highway, Shreveport, LA 71103, USA; judy.king@lsuhs.edu; 3 Department of Pharmacology, Edward Via College of Osteopathic Medicine, University of Louisiana Monroe, 4408 Bon Aire Drive, Monroe, LA 71203, USA; zelmageed@ulm.vcom.edu

**Keywords:** castration-resistant, prostate cancer, extra-virgin olive oil, nutraceutical, oleocanthal, progression, recurrence, SMYD2

## Abstract

**Simple Summary:**

The Mediterranean, extra-virgin-olive-oil-rich diet ingredient *S*-(-)-oleocanthal (OC) has emerged as a potential inhibitor for the growth and relapse of the most aggressive prostate cancer type. This effect is mediated through suppression of important enzyme, SMYD2, that drives the activation of several downstream protein effectors. OC treatments reduced SMYD2 downstream substrates, which are critical for prostate cancer growth and relapse. OC is more advantageous than other reported SMYD2 inhibitors because it has shown potent anticancer activity in animal models. OC’s anti-prostate-cancer effect was prominent compared with some standard drugs currently used to control prostate cancer. OC is a potential, novel natural compound appropriate for immediate use by prostate cancer patients and survivors as a nutraceutical or dietary supplement product.

**Abstract:**

Metastatic castration-resistant prostate cancer (mCRPC) is the most aggressive prostate cancer (PC) phenotype. Cellular lysine methylation is driven by protein lysine methyltransferases (PKMTs), such as those in the SET- and MYND-containing protein (SMYD) family, including SMYD2 methylate, and several histone and non-histone proteins. SMYD2 is dysregulated in metastatic PC patients with high Gleason score and shorter survival. The Mediterranean, extra-virgin-olive-oil-rich diet ingredient *S*-(-)-oleocanthal (OC) inhibited SMYD2 in biochemical assays and suppressed viability, migration, invasion, and colony formation of PC-3, CWR-R1ca, PC-3M, and DU-145 PC cell lines with IC_50_ range from high nM to low µM. OC’s in vitro antiproliferative effect was comparable to standard anti-PC chemotherapies or hormone therapies. A daily, oral 10 mg/kg dose of OC for 11 days effectively suppressed the progression of the mCRPC CWR-R1ca cells engrafted into male nude mice. Daily, oral OC treatment for 30 days suppressed tumor locoregional and distant recurrences after the primary tumors’ surgical excision. Collected OC-treated animal tumors showed marked SMYD2 reduction. OC-treated mice showed significant serum PSA reduction. For the first time, this study showed SMYD2 as novel molecular target in mCRPC, and OC emerged as a specific SMYD2 lead inhibitor. OC prevailed over previously reported SMYD2 inhibitors, with validated in vivo potency and high safety profile, and, therefore, is proposed as a novel nutraceutical for mCRPC progression and recurrence control.

## 1. Introduction

Prostate cancer (PC) is the most prevalent cancer among elderly men worldwide. It is the second leading cause of cancer death in American men. In 2020, the global incidence of PC was 29.3 per 100,000 [1]. One man out of every eight men in the United States is estimated to be diagnosed with PC during their lifetime [2]. About 268,490 new PC cases, which may lead to 34,500 deaths, occur each year [2]. About six PC cases are diagnosed in every 10 men 65 years old or older, while the disease is rare in men under the age of 40 [3].

The most common PC metastatic sites are bone (84%), distant lymph nodes (10.6%), liver (10.2%), and thorax (9.1%) [3,4]. Overall, 18.4% of patients have multiple metastatic sites involved [4]. Metastatic castration-resistant PC (mCRPC) is the most aggressive and recurrent PC phenotype [5]. In May 2020, the US Food and Drug Administration (FDA) approved two poly(adenosine diphosphate [ADP] ribose) polymerase (PARP) inhibitors for the treatment of mCRPC [6,7]. Rucaparib approved for the treatment of BRCA-mutant mCRPC after progression of the disease following prior androgen-receptor-directed therapy and taxane-based chemotherapy. Olaparib was approved for the treatment of homologous recombination repair gene-mutated mCRPC after progression following targeted treatment with enzalutamide (ENZ) or abiraterone acetate plus prednisone [6,7]. Despite the progress made toward androgen suppression, including the development of LH/FSH modulators, anti-AR small molecules, CYP17A and 27A1 inhibitors, chemotherapy, and immunotherapy, these treatments lack curative efficacy, especially in mCRPC patients, and have poor overall survival rates [5,6,7].

Cellular lysine methylation is driven by protein lysine methyltransferases (PKMTs), including those of the SET- and MYND-containing protein (SMYD) family. SMYD PKMTs are critical for gene regulation, chromatin remodeling, transcription, signal transduction, cell cycle control, and DNA damage response. The SET (suppressor of variegation, enhancer of zeste, trithorax) and MYND (myeloid-nervy-DEAF1) domain-containing protein 2, SMYD2, has emerged as an oncogenic protein lysine methyltransferase that methylates the histones H3K4 and H3K36 correlated with epigenetic regulation [8]. Apart from histone methylation, SMYD2 is implicated in the methylation of several important, non-histone protein substrates such as the tumor suppressor p53, retinoblastoma tumor suppressor (Rb), estrogen receptor α (ERα), PARP-1, and echinoderm microtubule-associated proteins, such as the 4-ALK receptor tyrosine kinase fusion gene, heat shock protein 90, and β-catenin [9]. Lysine methylation by SMYD2 modifies protein substrates’ bulkiness, hydrophobicity, and molecular recognition by methyllysine readers. Methyllysine-containing proteins are recognized by downstream effectors possessing methyllysine reader domains, conferring their biological effects. Given the implications of methylation of non-histone proteins as some of the leading key players in the network of post-translational modifications, which appear to be crucial for tumorigenesis, SMYD2 has been explored as a putative oncogenic driver [10,11,12,13,14,15,16,17,18]. Accumulating evidence has highlighted the amplification of SMYD2 in multiple malignancies, including esophageal squamous cell carcinoma, pancreatic cancer, pediatric acute lymphoblastic leukemia, breast cancer, teratocarcinoma, and gastric, ovarian, head and neck, and colorectal cancers [10,11,12,13,14,15,16,17,18]. SMYD2 amplification is a poor prognosis marker for low overall patient survival. SMYD2 plays a pivotal role in cancer through the induction of cell proliferation, migration, and invasion, as well as inhibition of apoptosis [8]. A few synthetic, small-molecule SMYD2 inhibitors have been developed with impressive in vitro potency and selectivity but most of them did not show a good in vivo activity profile or had no reported in vivo efficacy, and, therefore, none of them earned FDA approval [19,20,21]. The literature has not addressed the role of SMYD2 in PC, but the closely related SMYD family member SMYD3 proved a valid molecular target in PC since it modulates transcriptional and key signaling pathways and orchestrates multiple oncogenic inputs [22].

*S*-(-)-oleocanthal (OC) is a distinctive, monophenolic secoiridoid constituent of extra-virgin olive oil (EVOO), possessing documented anti-inflammatory, antioxidant, antimicrobial, neuroprotective, and anticancer activities [23]. The Beauchamp group exhibited a pivotal lead by identifying OC as the pungent and irritant, potent, anti-inflammatory EVOO ingredient able to perform activity comparable to the non-steroidal, anti-inflammatory drug ibuprofen by inhibiting COX-1 and COX-2 activity [24]. There is substantial evidence validating OC’s anti-inflammatory activity by modulation of IL-6 and 5-lipoxygenase secretion [25]. Emerging evidence reported several mechanisms in which OC induces apoptosis and inhibits migration, angiogenesis, and metastasis of breast, hepatocellular, melanoma, non-melanoma skin, and colorectal cancers [23]. OC’s anticancer properties are facilitated by suppressing the mesenchymal–epithelial transition factor (c-MET) receptor tyrosine kinase [26] and modulating its downstream signaling pathways [23], cyclooxygenase-2 (COX-2) [26], phosphorylated mechanistic target of rapamycin (mTOR) [27], and signal transducer and activator of transcription 3 (STAT3) levels [28]. In addition, this study team showed earlier that oral OC treatments prevented both triple-negative and luminal B breast cancer recurrences [29,30]. There are no current comprehensive reports on the anti-PC effects of OC that include consideration of in vivo progression and recurrence suppressions, along with its molecular target(s). Considering the importance of the pleotropic impacts of SMYD2 on cancer biology [15,16,17,18] and the hypothesis that targeting SMYD2 may impair the PC recurrence, the current study reports the novel role of SMYD2 in mCRPC pathogenesis. In addition, the study highlights the ability of OC to control mCRPC progression and recurrence by targeting SMYD2 in vitro and in vivo using nude mouse xenograft models. The taxane microtubule depolymerization stabilizers paclitaxel (PTX) and docetaxel (DTX), the FDA-approved, selective, second-generation androgen receptor (AR), competitive antagonist enzalutamide (ENZ), and cisplatin (CPT) were used as standard drug controls for the in vitro studies. These four chemotherapies or hormone therapies are among the standard therapeutic options for the control of mCRPC [31].

## 2. Materials and Methods

### 2.1. Chemicals and Reagents

All chemicals were purchased from VWR International (Suwanee, GA, USA) unless otherwise stated. *S*-(-)-oleocanthal (OC) was extracted from EVOO (The Governor, Corfu, Greece), and a purity of >99% was established based on q^1^H NMR analysis [32]. OC extraction, purification, and analysis followed the liquid–liquid extraction methodology described earlier by this study team [32].

### 2.2. Cell Lines and Culture Conditions

The American Type Culture Collection (ATCC, Manassas, VA, USA) was the commercial source for the human PC cell lines DU-145, PC-3, and PC-3M. Dr. Zakaria Abd Elmageed, Department of Pharmacology, Edward Via College of Osteopathic Medicine, Monroe, LA, USA, generously provided the CWR-R1ca cells [33]. Cells cultured in Roswell Park Memorial Institute (RPMI-1640) or Dulbecco’s Modified Eagle’s medium (DMEM) media were supplemented with 10% FBS (fetal bovine serum), penicillin G (100 U/mL), and 100 ng/mL of the aminoglycoside antibiotic streptomycin. Cells were maintained with 5% CO_2_ in a humidified incubator at 37 °C. Cells were washed with Ca^2+^ and Mg^2+^-free phosphate-buffered saline (PBS) and incubated at 37 °C in 0.05% trypsin possessing 0.02% ethylenediaminetetraacetic acid (EDTA) for 3–5 min for subculturing.

### 2.3. Experimental Treatments

To prepare 25 mM stock solution, OC was dissolved in sterile dimethyl sulfoxide (DMSO). This OC stock solution was used to prepare various treatment concentrations for various experiments. The final concentration of DMSO was maintained at the same level in all treatment groups within a given experiment and never exceeded 0.1% in sterile PBS.

### 2.4. Cell Viability Assay

About 1 × 10^4^ cells/well, six replicates per treatment group, were seeded into 96-well plates in 10% FBS RPMI-1-640 media and left overnight to attach. Cells were divided into different treatment groups and exposed to respective control or experimental treatments the next day with various OC or vehicle control concentrations for 24 or 48 h in media. 3-(4,5-dimethylthiazolyl2)-2,5-diphenyltetrazolium bromide (MTT) was applied to quantify the viable cell number at the end of experiment [34,35]. A final concentration of 1.0 mg/mL MTT was added to each well. Following a 4 h incubation at 37 °C, media were removed, and the formazan crystals solution in DMSO was added at 100 µL per well. The optical density was measured at 570 nm on a microplate reader (BioTek, Winooski, VT, USA) for quantification using a standard calibration curve, as previously described [25,32].

### 2.5. CRISPR-Cas9 SMYD2 Knockout (KO)

The executed procedure followed the manufacturer’s guidelines (Santa Cruz Biotechnology, Dallas, TX, USA). In brief, in a 6-well tissue culture plate, we seeded 1.5 × 10^5^–2.5 × 10^5^ cells in 2 mL of standard growth medium per well 24 h prior to transfection. Cells were grown to 40–80% confluency. Healthy and subconfluent cells are required for a successful KO experiment. About 1 µg of SMYD2 plasmid DNA was diluted into plasmid transfection medium (Santa Cruz Biotechnology, Dallas, TX, USA, sc108062) to bring the final volume to 150 µL and was maintained for 5 min at room temperature (RT) (Solution A). Next, 10 µL of diluted UltraCruz^®^ Transfection Reagent (sc395739) was added with enough plasmid transfection medium to bring the final volume up to 150 µL and left for 5 min at RT (Solution B). Further, the plasmid DNA solution (Solution A) was directly added dropwise to the dilute UltraCruz^®^ Transfection Reagent (Solution B). The mixture was vortexed immediately and incubated for 20 min at RT. Prior to transfection, the cells were washed with PBS and fresh, antibiotic-free growth medium. The 300 µL plasmid DNA/UltraCruz^®^ Transfection Reagent Complex (Solution A + Solution B) was added dropwise to each well and gently mixed by swirling the plate. Cells were incubated for 24 h under conditions normally used to culture. No media replacement was necessary during the first 24 h. Media were replaced as needed over the 24–72 h post transfection. After incubation, successful transfection of CRISPR/Cas9 KO plasmid was confirmed by Western blotting of wild CWR-R1ca versus CWR-R1ca-KO cells using SMYD2 polyclonal antibody purchased from ProteinTech (Rosemont, IL, USA) at a dilution of 1:1000.

### 2.6. Wound-Healing Assay

The mCRPC CWR-R1ca and PC-3 cells were plated in sterile, 24-well, flat-bottom plates (3 replicates/group). Cells were allowed to form a subconfluent monolayer per well overnight. Wounds were inflected in each cell monolayer using a sterile pipette tip (200 µL). Media were then removed and, using sterile PBS, cells were washed twice. Cells were then incubated in 0.5% serum-containing culture media, to which various OC treatments were added. Cells were incubated for 24 h or until the vehicle control (VC) wells’ wound closed. The media were then removed and the cells washed with sterile, precooled PBS and fixed with absolute ethanol and stained with Giemsa. The healing of each wound was visualized at 0 and 24 h, or until the full closure of the VC wound, using Nikon ECLIPSE TE200U microscope (Nikon Instruments Inc., Melville, NY, USA). Digital images of each wound were captured and travel distance determined by comparing the wound width at 24 h or ending the experiments’ hours with the wound width at the start of treatment (zero time). The obtained % migration was calculated by setting the gap width at t0 as 100%. Each experiment was conducted in triplicate to confirm reproducibility.

### 2.7. Colony Formation Assay

The mCRPC CWR-R1ca and PC-3 cells were seeded in 12-well plates (1000 cells/well). Cells were treated with OC at different concentrations after 24 h. Media were replaced every 72 h with or without treatments. Experiment continued until the control had distinct colony formation, mostly within 10–14 days. Each treatment colony was stained with crystal violet and photographed. The number of colonies was scored by CFU Scope quantification software [36]. Results were expressed as the number of colony-forming cells per well in percentage and normalized to the control (VC represented 100%).

### 2.8. Transwell Migration Assay

The Radius™ 96-Well Cell Migration Assay kit was used (Cell BioLabs, San Diego, CA, USA). CWR-R1ca cells were seeded in each well’s upper chamber (5 × 10^5^ cells/well), containing either vehicle control or different OC concentration treatments. Ten percent serum-containing media was added to each lower chamber well as a chemoattractant. After 24 h, all chambers were washed with PBS and fixed with cold methanol on ice for 10 min. All chambers were then stained with Gemsia and washed with water. Finally, all chambers were subjected to imaging using inverted Nikon microscope. For each chamber, multiple images were captured randomly and further quantified. Results were expressed as the percentage of migration and normalized to vehicle control treatments, which assumed 100% migration.

### 2.9. Invasion Assay

The BD BioCoat™ BD Matrigel™ Invasion kit (BD Biosciences, San Diego, CA, USA) was used. CWR-R1ca cells were seeded in each upper chamber (5 × 10^5^ cells/well) and precoated with Matrigel with either the vehicle control or different OC treatments. Ten percent of serum-containing media was added to the lower chambers as chemoattractant. After 24 h, all the chambers were washed with PBS and fixed with cold methanol on ice for 10 min. All chambers were then stained with Gemsia and washed with water. Finally, all chambers were imaged using inverted Nikon microscope (Nikon Instruments Inc., Melville, NY, USA). For each chamber, multiple images were randomly captured and further quantified. Results were expressed as the percentage migration normalized to the VC, which assumed 100% migration.

### 2.10. Western Blot Analysis

About 1 × 10^6^ CWR-R1ca and PC-3 cells were plated in culture plates (10 cm) in RPMI 1640 containing 10% FBS. Cells were allowed to attach overnight. Cells were washed with PBS and treated with media containing VC or various OC concentrations for 48 h. Cells were then collected and washed twice with sterile, cold PBS. Cells were then resuspended and lysed at 4 °C for 30 min in radioimmunoprecipitation assay (RIPA) buffer (Qiagen Sciences Inc., Valencia, CA, USA). Cell lysates were centrifuged for 10 min at 14,000× *g* and the supernatants stored at −80 °C as whole-cell extracts. For collected mouse tumor samples, tumor tissue samples were collected at the primary tumor surgical excision time or at sacrifice and immediately stored at −80 °C until protein extraction. Collected tumor samples were homogenized by electric homogenizer in RIPA buffer. Each sample protein concentration was calculated by the Pierce BCA Protein Assay (Thermo Fisher Scientific Inc., Rockford, IL, USA). Sample proteins were separated on 10% sodium dodecyl sulfate-polyacrylamide gel electrophoresis gels and transferred to polyvinylidene difluoride membranes. Membranes were blocked with EveryBlot blocking buffer (Biorad, Hercules, CA, USA) and incubated with specific primary antibodies. The corresponding horseradish peroxidase-conjugated secondary antibodies were used against each primary antibody. The Chemi-Doc XRS chemiluminescent gel imaging system was used to detect and analyze sample proteins by applying Image Lab software (BioRad, Hercules, CA, USA). The β-tubulin visualization was used to ensure equal sample loading to each lane. Each experiment was repeated three times, and representative images were used in results figures.

### 2.11. Lentivirus Transduction-Aided Luciferase Labeling of CWR-R1ca Cells

CWR-R1ca cells were seeded into 12-well plates. When the cells reached 60% confluency, lentiviral particles carrying luciferase (Kerafast, Boston, MA, USA) were transduced into the cells. Briefly, the lentivirus vector was added to OptiMEM^®^-reduced serum media (1.5 μL/100 μL) and mixed very gently on ice. Later, the media were aspirated, and cells were washed with PBS. Then, 100 μL of OptiMEM^®^ media, with or without viral particles, was added to each well and incubated for 18 h. Later, the media were aspirated and replaced with complete serum media for 2 days. Next, 0.15 μg/mL of puromycin was used for selection and maintenance of cells expressing the luciferase. Fresh media containing puromycin replaced the old media every other day. Cellular luciferase activity was measured by adding 25 μL of XenoLight D-luciferin K^+^ salt bioluminescent substrate (PerkinElmer, Waltham, MA, USA) at a dose of 150 mg/kg in PBS into each well and incubated for 5 min at RT [37]. Cells were then imaged using IVIS bioluminescence imaging system (PerkinElmer’s IVIS^®^ Lumina III imaging platform).

### 2.12. Animal Models and Treatment

Mice were acclimated and maintained under clean room conditions in sterile filter top cages in the University of Louisiana at Monroe (ULM) vivarium using AlphaDri bedding. Mice were housed at 25 °C and 55–65% relative humidity with high-efficiency particulate air-filtered ventilated racks with 12 h light/dark cycle for a week before the study’s experiments. Study mice had open access to water and pelleted rodent 5% fat content diet chow (Cat #7012, Envigo-Teklad, Madison, WI, USA). Animal procedures were preapproved by the ULM Institutional Animal Care and Use Committee (IACUC), protocol number 21MAY-KES-01, and all experimentations were conducted in strict accordance with NIH-guided good animal practices. The tumor volume (V) in each mouse was monitored and calculated by the formula V = L/2 × W^2^, where L is the tumor length, and W is the width. Mice clinical health profiles (food and water consumption, defecation, urination, and physical activity) and body weights were carefully monitored on a daily basis over the study course.

#### 2.12.1. OC Suppressed mCRPC CWR-R1ca-Luc Cell Progression In Vivo in Nude Mouse Model

The male athymic nude mice (Foxn1^nu^/Foxn^1+^, 4–5 weeks) were acquired from Envigo (Indianapolis, IN, USA). Mice were housed in group cages, 5 in each. Live animal bioluminescence imaging was conducted weekly, after anesthetizing mice with 2% isoflurane, on IVIS Lumina series III (Perkin Elmer) imaging system 20 min after 150 mg/kg XenoLight D-luciferin K^+^ salt bioluminescent substrate (PerkinElmer) intraperitoneal (i.p.) injection. Emitted photons by CWR-R1ca-Luc luciferase-expressing cells, which transmitted through animal tissues, were quantified using the Living Image software program (PerkinElmer). Each mouse was injected at the suprascapular region with 2 × 10^6^ CWR-R1ca-Luc cells. Mice were randomized to two groups, 5 in each, once their tumors were palpable and reaching 200–300 mm^3^. These groups were (i) placebo control and (ii) 10 mg/kg OC administered orally daily by gavage. OC was formulated as powder formulation (PF) for oral dosing, as previously described by this study team [35]. Briefly, 10 mg OC adsorbed on Aerosil 200 (3 mg) then mixed with Mg stearate (0.5 mg) and Na lauryl sulfate (2 mg). About 84.5 mg lactose was then added, and all ingredients were uniformly blended to afford OC-PF [35]. This mixture was then passed over a 40-mesh screen to afford OC-PF [35]. Mice were treated orally with either OC-PF or placebo control for 11 days. OC-PF was orally administered by using a flexible plastic (2 mm diameter) gavage tube with stainless steel bite protector, 18 gauge, 3.81 cm long.

#### 2.12.2. Oral Treatments with OC-PF Effectively Suppressed mCRPC CWR-R1ca-Luc Cells’ Recurrence in Nude Mouse Model after Primary Tumor Surgical Excision

The primary tumors of the mice in the previous growth study were surgically excised and used for recurrence study. Prior to the surgical excision procedure, mice were anesthetized by i.p. injection of ketamine/xylazine mixture (100 mg/kg + 15 mg/kg, respectively) [25]. Nearly 15–20 min after injecting the anesthesia, animal reflexes were tested by gently tapping the hind legs with a sterile syringe needle to confirm their full anesthesia. The primary tumors were aseptically surgically excised, and each wound stitched. A 1 mg/kg amount of ketoprofen was used 12 h before and after the surgery for effective analgesia. Bupivacaine (0.25%, 1–2 drops) was used topically twice daily at the excision wound site to prevent local infiltration along the surgery site during closure with a maximum dose of 2 mg/kg. Collected primary tumors were stored at −80 °C. Mice continued to be subjected to daily, oral placebo control or 10 mg/kg OC-PF treatments for 30 days. Mice were subjected to weekly live imaging. Mice were monitored daily for new tumors, body weights, and general health characters. Mice were regularly observed to assure post-surgery wound healing. The results were presented as average ± SD. All mice were sacrificed at the end of the experiments. Fresh blood samples were collected in microtainer tubes and immediately centrifuged at 4 °C for 10 min at 13,000 rpm to prepare serum samples, which were stored at −80 °C until PSA quantification. Collected tumors were stored at −80 °C until total protein was extracted for Western blot analysis. Collected organs were imaged and stored in 10% formalin for 48 h, followed by storing in 70% ethanol until histology processed [25].

### 2.13. Quantification of PSA Serum Levels Using ELISA Kit

Mice serum PSA levels were measured using an enzyme-linked immunosorbent assay (ELISA) kit (MYBIOSOURCE, San Diego, CA, USA) according to manufacturer protocol. Briefly, 50 µL sample was added to each well, followed by 100 µL HRP-conjugate reagent, and incubated for 1 h at 37 °C. Then, 50 µL of each chromogen solution, A and B, was added to each well and incubated for 15 min. Later, the reaction was stopped by adding 50 µL of stopping solution. OD was read at 450 nm using an ELISA plate reader (BioTek, Winooski, VT, USA).

### 2.14. Immunohistochemistry (IHC) Study

The IHC slides prepared from paraffin-embedded tumor tissue samples were sectioned to 5 μm thick sections at AML Laboratories (Jacksonville, FL, USA). IHC protocol followed our earlier studies [35]. Following the de-paraffinization in xylene and graded ethanol, sections were boiled in citrate buffer (10 mM sodium citrate, pH 6) for 20 min and then permeabilized in TBST solution for 15 min at 25 °C. Sections were then stained with the primary antibodies of ki67 (Cat #9129, 1:200, Cell Signaling, Boston, MA, USA) or CD-31 (Cat #3528, 1:200, Cell Signaling) and further diluted in blocking solution for 24 h at 4 °C. On the following day, sections were washed, having been stained with the secondary antibodies for 1 h prior. At the experiment end, slides were mounted. All images were captured at the Research Core Facility, LSUHSC, Shreveport, LA, USA, with 10× magnification using Olympus iXplore CSU W1 spinning disk confocal microscope (Center Valley, PA, USA).

### 2.15. Hematoxylin and Eosin Y (H&E) Staining

Tumor samples were fixed in 10% neutral buffered formalin for 48 h. This was followed by transferring to 70% ethanol, and paraffin embedding was processed. Paraffin-embedded tumor blocks were sectioned into 5 µm sections using a Leica RM2035 microtome by AML laboratories (Augustine, FL, USA). Sections were mounted on positively charged slides, dewaxed in xylene, rinsed with alcohol, rehydrated in water, and, finally, tumor slides were stained with H&E [25,34].

### 2.16. Analysis of Clinical SMYD2 Gene Expression Data

The TCGA gene expression data were analyzed using UALCAN, which is a publicly available web tool able to perform in-depth analysis [38,39]. The mRNA expression pattern of SMYD family was analyzed. The gene expression profiling interactive analysis (GEPIA) was used to explore RNA sequence expression difference between normal and cancer samples [40]. GEPIA was used for differential expression analysis comparison of SMYD family in various cancers versus normal organ tissues [41]. The cBioPortal for Cancer Genomics is a widely used web platform for exploring, visualizing, and analyzing multidimensional cancer genomics datasets [42,43,44]. cBioPortal was applied in this study to explore expression of SMYD2 with defined parameter settings.

### 2.17. Statistics

Data analysis was performed using GraphPad Prism software, version 8.4.3. (La Jolla, CA, USA). Results were presented as mean ± standard deviation (SD) for continuous variables. Differences among various treatment and control groups in the animal study were determined by paired Student’s *t*-test, and *p*-value implications were: * *p* < 0.05, ** *p* < 0.01, and *** *p* < 0.001.

## 3. Results

### 3.1. Prognosis Analysis of SMYD Family mRNA Expression in Prostate Cancer Patients

The cBioPortal database was utilized to understand the link between the SMYD family and PC prognosis. Regarding overall gene alteration, in the three different study cohorts, SMYD2 and SMYD3, out of the five SMYD family members, had the biggest role in amplification, deep deletion, and missense mutations, unlike the other SMYD family members, which contributed minor roles (Figure S1A). Excluding SMYD4, the rest of the SMYD family members were strongly linked with both primary and metastatic PC (Figure S1B). SMYD2 was observable at high Gleason scores (Figure S1C). Unlike the rest of the SMYD members, only SMYD2 expression was associated with high Gleason scores (Figure S1C). Similarly, SMYD2 was associated with high radical prostatectomy Gleason score PC, unlike other SMYD family members (Figure S1D). Overall, patient-based findings clearly showed that SMYD family members are clinically relevant to PC progression. Out of the SMYD family members, SMYD2 is the highest prognostic marker associated with poor PC prognosis, aggressive progression, and metastasis profiles.

### 3.2. Prognostic Analysis of SMYD2 mRNA Expression in PC Patients

The Cancer Genome Atlas (TCGA) was analyzed through UALCAN’s GEPIA to investigate the differential expression pattern of SMYD2 in various types of cancer and, more specifically, PC types. Findings showed that SMYD2 is overexpressed in many cancer types, including breast, lung, liver, and prostate adenocarcinoma (Figures S2A and S3A). Further exploration also confirmed that, in one cohort, SMYD2 was more significant (*p* = 1.46 × 10^−11^) in primary PC (*n* = 497) compared to in normal tissue (*n* = 52) samples (Figure S2B). Similar observations were noted in another cohort using GEPIA 2, validating the existence of SMYD2 overexpression in patient PC samples (*n* = 492) versus normal tissue (*n* = 152) samples (Figure S3B). Further comparison among normal, primary, and metastatic tumors showed the significant overexpression of SMYD2 in metastatic PC (Figure S3C). Interestingly, TCGA sample analysis showed the high SMYD2 overexpression in nodal prostate metastasis N1 (*n* = 79) compared to normal tissues (*n* = 52) and N0 samples (*n* = 345) (Figure S3D). Regarding the PC patients’ Gleason score, SMYD2 was observable at a Gleason score of 6 and higher (Figure S3C).

### 3.3. Exploration of SMYD2 Expression Pattern in PC and Non-Tumorigenic RWPE-1 Prostatic Epithelial Cells

**Figure 1:**
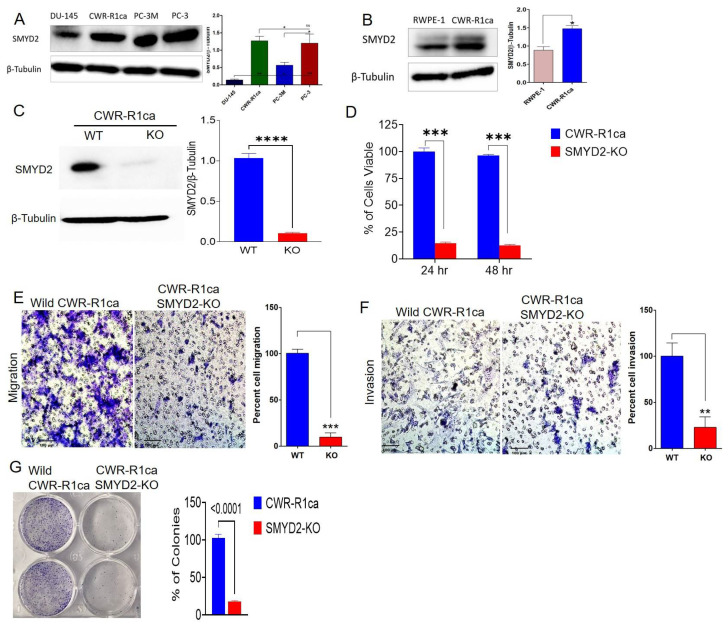
The SMYD2 expression pattern in PC and non-tumorigenic prostate cell lines and its critical contribution to the mCRPC CWR-R1ca cells’ proliferation, migration, invasion, and colony formation. (**A**) Expression pattern of SMYD2 in mCRPC and AI PC cell lines. (**B**) Comparison of the expression levels of SMYD2 in the non-tumorigenic RWPE-1 prostatic epithelial cells versus the mCRPC CWR-R1ca cells. Immunoblots show the upregulation of SMYD2 in CWR-R1ca cells compared to RWPE-1 cells. Bar graph represents the densitometric analysis of SMYD2 expression level normalized to β-tubulin in both cells. (**C**) CRISPR-Cas9-aided successful SMYD2 knockout evidenced by comparing the Western blot of the wild type versus SMYD2-KO CWR-R1ca cell lysates. All raw immunoblots are included in Figure S7. (**D**) Comparison of the viability of the wild CWR-R1ca versus CWR-R1ca-KO cells in MTT assay over 24 and 48 h. (**E**) Comparison of the migration ability of the wild CWR-R1ca versus CWR-R1ca-KO cells in wound-healing assay. (**F**) Comparison of the invasiveness of the wild CWR-R1ca versus CWR-R1ca-KO cells in Matrigel invasion assay. (**G**) Comparison of the colony formation of the wild CWR-R1ca versus CWR-R1ca-KO cells in colony formation assay. SMYD2 knockout conferred significant viability, migratory, invasiveness, and colony formation suppression compared with the wild CWR-R1ca cells. Vertical bars indicate the percentage relative to that in the vehicle control. Data represent the mean ± SD (*n* = 3); Student’s *t*-test; * *p* < 0.05, ** *p* < 0.01, *** *p* < 0.001, **** *p* < 0.0001, ns refers to non-statistical significance at *p* < 0.05 relative to control cells.

This study explored the expression level of SMYD2 in diverse PC cell lines. Western blotting analysis indicated that SMYD2 excessively dysregulated the highly recurrent castration-resistant PC (mCRPC) CWR-R1ca cells, as well as the androgen-independent PC-3, PC-3M, and DU-145 PC cells (Figure 1A). Western blotting comparison of the expression level of SMYD2 indicated 1.8-fold the SMYD2 expression level in the mCRPC CWR-R1ca cells compared to the non-tumorigenic RWPE-1 prostatic epithelial cells (Figure 1B). The mCRPC cell lines CWR-R1ca and PC-3 expressed the highest SMYD2 levels, followed by the AI PC-3M cells, which expressed half the SMYD2 level of the mCRPC cells. DU-145 cells showed the lowest SMYD2 expression level compared to other PC cell lines (Figure 1A).

### 3.4. SMYD2 Is Essential for mCRPC Cell Survival and Motility

To determine whether SMYD2 is essential for the viability and motility of the mCRPC cells, a CRISPR-Cas9 methodology was successfully used to knock out (KO) SMYD2 in the CRPC CWR-R1ca cells (CWR-R1ca-KO), evidenced by comparing the wild-type and KO cells by Western blotting (Figure 1C). SMYD2 KO significantly reduced (>90%) the CWR-R1ca cells’ viability over 24 and 48 h post transfection (Figure 1D). Cancer cells possess broad-spectrum migration and invasion mechanisms, which aid the motility and subsequent metastasis to distant organs [45]. Results showed that the CWR-R1ca-KO cells more significantly suppressed the migration and invasion ability of cells over 24 h (Figure 1E,F, respectively) than the wild-type CWR-R1ca cells. Meanwhile, the colony formation assay is an in vitro alternative cell survival assay based on the ability of a single cell to adhere and form a viable colony. It is a good in vitro experimental approach to mimic the in vivo recurrence in which the circulating tumor cell adheres to a distant organ, forms a colony, and develops subsequent, metastatic foci [46]. SMYD2-knockout-I CWR-R1ca cells (CWR-R1ca-KO) significantly suppressed the colony formation ability compared to the wild-type cells (Figure 1G), allowing us to better understand the role of SMYD2 in tumor cell colony formation. Collectively, these results indicate that SMYD2 is a major contributor to the cell viability and motility of the CRPC, as represented by the CWR-R1ca cells.

### 3.5. OC Inhibited SMYD2 Monomethylation in Biochemical Assays

The methyltransferase activity assays performed at the Eli Lilly Open Innovation Drug Discovery (OIDD) Program. The assays included the monitoring of ^3^H-labeled methyl groups incorporated into a peptide residue, 361–380 of p53, using scintillation proximity assay [47]. A single 10 µM dose of OC inhibited 90.8% of SMYD2 ability in monomethylate p53 Lys^370^ [47].

### 3.6. OC Treatments Selectively Suppressed the Viability of PC Cells by Reducing SMYD2 Expression but Did Not Adversely Affect the Non-Tumorigenic RWPE-1 Prostatic Epithelial Cells’ Viability at Therapeutic Doses

**Figure 2:**
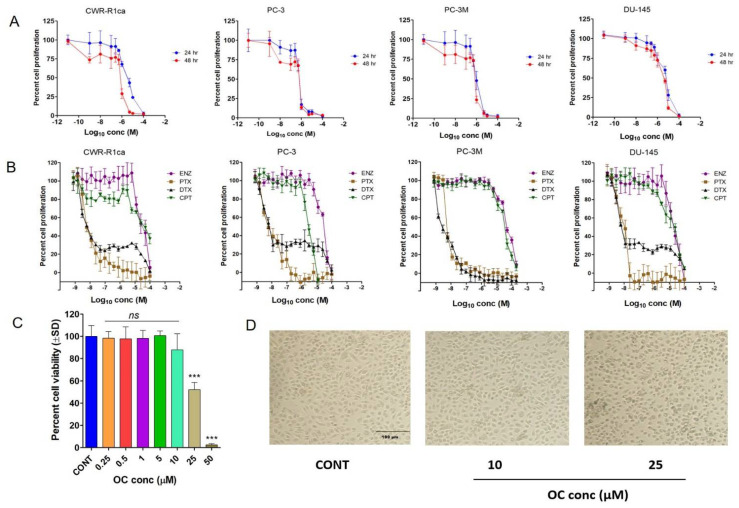

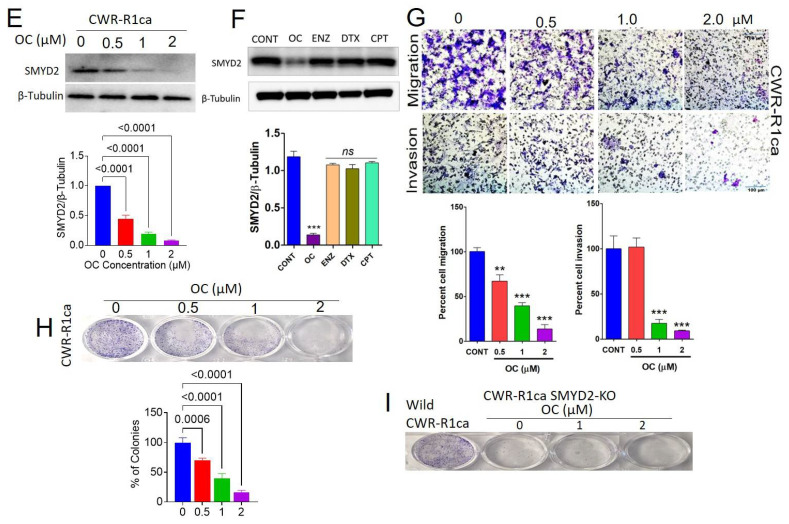
Oleocanthal treatments effectively inhibited the in vitro PC cell proliferation, migration, invasion, and colony formation. (**A**) The effect of OC treatments on the growth of CWR-R1ca, PC-3, PC-3M, and DU-145 cells over 24 h and 48 h treatment periods. (**B**) The effects of enzalutamide, paclitaxel, docetaxel, and cisplatin on the growth of PC cell lines over 24 h and 48 h treatment periods. (**C**) Effects of OC treatment on the viability of the non-tumorigenic immortalized prostatic epithelial RWPE-1 cells. Bar graph represents the mean cell viability (±SD) at indicated concentrations relative to untreated control cells. (**D**) Microscopic photographs showing healthy cells in control and at 10 µM OC, while showing a significant cell toxicity at 25 µM OC. (**E**) Western blot showing the suppressive effect of OC treatments on the SMYD2 expression in the mCRPC CWR-R1ca cells over 48 h culture period. (**F**) Comparison of the SMYD2 expression level suppression of OC versus ENZ, DTX, and CPT anticancer therapeutics in CWR-R1ca cells. Immunoblots show the effective downregulation of SMYD2 in CWR-R1ca cells treated with 2 µM OC. Meanwhile, treatments with standard anticancer drugs ENZ (100 µM), DTX (5 nM), and cisplatin (30 µM) did not significantly affect the expression level of SMYD2 in comparison to untreated control cells. Bottom bar graph represents the densitometric analysis of SMYD2 expression levels normalized to β-tubulin in control and treated cells. All raw immunoblots are included in Figure S7. (**G**) The effect of OC treatments on the migration and invasion of CWR-R1ca cells over 24 h treatment period. (**H**) The effect of OC treatments on the colony formation of wild CWR-R1ca cells. (**I**) The effect of OC treatments on the colony formation of CWR-R1ca-KO cells. Vertical bars indicate the percentage relative to that in the vehicle control. Data represent the mean ± SD (*n* = 3); one-way ANNOVA; ** *p* < 0.01, *** *p* < 0.001; ns refers to non-statistical significance at *p* < 0.05 relative to control cells.

Earlier studies reported the activity of OC against PC-3 PC cell proliferation and migration by targeting c-MET receptor tyrosine kinase [48]. The antiproliferative activity of OC was evaluated against the mCRPC CWR-R1ca and PC-3 cells, as well as the androgen-independent/indifferent (AI) PC-3M PC cells (Figure 2A and Table 1). OC treatments showed significant dose- and time-dependent inhibition of the proliferation of all PC cells with an 0.58–3.59 μM IC_50_ range in MTT assay (Figure 2A and Table 1). The in vitro antiproliferative activity of OC against PC cell lines was compared with a panel of standard anti-PC chemotherapies or hormone therapies, including ENZ, PTX, DTX, and CPT, over a 48 h treatment period. The taxanes PTX and DTX were the most potent against PC cell lines, with IC_50_ in a low nM range (Figure 2B and Table 1). ENZ and CPT were far less active with IC_50_ at a high µM range. Meanwhile, OC only affected the non-tumorigenic RWPE-1 prostatic epithelial cell viability at high µM doses, with IC_50_ in the range of 25 µM (Figure 2C,D). This profile indicated high OC selectivity for malignant versus non-tumorigenic cells, which was adversely affected at 10-fold OC therapeutic concentration, implying a potential high selectivity and safety profile. Treatment of CWR-R1ca cells with 0.5–2 μM OC for 48 h notably suppressed the SMYD2 expression, as evidenced by Western blotting assessment (Figure 2E), further validating SMYD2 as a prospective molecular target in these cells. Comparison of the SMYD2 expression level suppression of OC versus ENZ, DTX, and CPT anticancer therapeutics in CWR-R1ca cells showed the effective downregulation of SMYD2 in CWR-R1ca cells in response to 2 µM OC treatment. Meanwhile, treatments of CWR-R1ca cells with concentrations slightly above the IC_50_ values of the standard anticancer drugs ENZ (100 µM), DTX (5 nM), and cisplatin (30 µM) did not significantly affect the expression level of SMYD2 in comparison to untreated control cells (Figure 2F). This clearly validated the selective OC SMYD2 expression suppression effects in mCRPC cells.

### 3.7. OC Treatments Suppressed the Migration and Invasion of the CRPC CWR-R1ca Cells

The Radius™ migration kit was used to assess the antimigratory activity of OC against CWR-R1ca cells (Figure 2G). OC 0.5, 1.0, and 2.0 µM treatments showed significant and dose-dependent inhibition CWR-R1ca cell migration over the 24 h treatment period. Further, the BD Biocoat Matrigel invasion chamber, containing an 8 µm pore size PET membrane with a thin-layer Matrigel basement membrane matrix, was used to assess the anti-invasive effect of OC against CWR-R1ca cells. The number of migrated/invaded cells in each well was counted visually. Each experiment was performed in triplicate for reproducibility and statistical relevance confirmation. OC treatments significantly and dose-dependently inhibited CWR-R1ca cells’ invasiveness (Figure 2G).

### 3.8. OC Treatments Inhibited the Colony Formation of the Wild but Not the SMYD2-KO CWR-R1ca Cells

The CWR-R1ca-KO cells showed reduced colony formation after the KO of SMYD2 in these cells. The effect of OC treatments on the colony formation of the wild CWR-R1ca and CWR-R1ca-KO PC cells was assessed. The number of colonies in each well was manually counted. Each experiment was performed in triplicate for reproducibility and statistical relevance confirmation. OC treatments dose-dependently suppressed the colony formation of the wild-type CWR-R1ca cells. In contrast, the colony formation ability of the CWR-R1ca-KO cells was notably suppressed by SMYD2 KO. The CWR-R1ca-KO cells’ colony formation was not affected much by OC treatments (Figure 2H,I).

### 3.9. Oral OC-PF Treatments Suppressed the Progression of CWR-R1ca-Luc Cells Engrafted into Nude Mice

**Figure 3:**
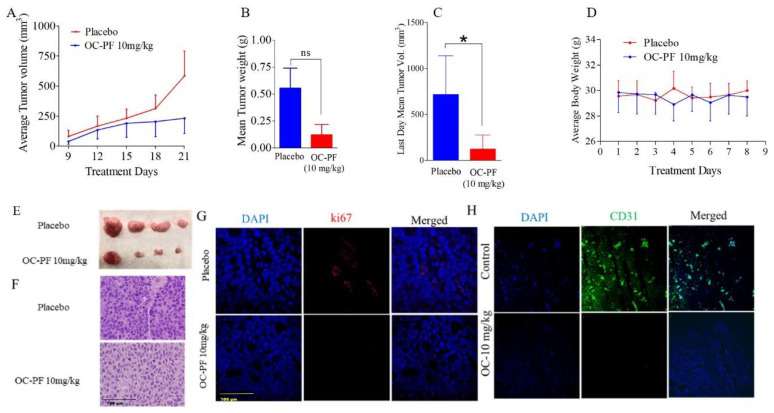
In vivo mCRPC suppressive effects of daily OC 10 mg/kg oral treatments against CWR-R1ca-Luc cells engrafted in male nude mice. (**A**) Comparative monitoring of the mean CWR-R1ca-Luc cells tumor volume for the OC-PF-treated daily oral 10 mg/kg mice versus the placebo control-treated group over the experiment duration (11 days). Points represent the mean tumor volumes, and error bars represent the SED for each experimental group. (**B**) The mean tumor weights in OC-PF-treated versus placebo control-treated mice at the experiment end. (**C**) Comparison of the mean tumor volume for OC-PF-treated versus placebo control-treated mice on the last study day. (**D**) Mice body weights monitoring over the experiment course. Points represent the mean body weight for animals in each group. (**E**) Representative primary tumors of each experimental group collected after surgical excision surgeries. Top row is the collected placebo control-treated primary tumors. The bottom row is the OC-treated collected primary tumors. Error bars indicate SD. (**F**) Histopathological evaluation of OC-PF treatment versus placebo control at 20× magnification. (**G**) Comparison of OC-PF-treated versus placebo control-treated primary tumor immunofluorescence expression of ki67. (**H**) Comparison of OC-PF-treated versus placebo control-treated primary tumors immunofluorescence expression of CD31. * *p* < 0.05 for statistical significance compared to the placebo control group. ns refers to non-statistical significance at *p* < 0.05 relative to control cells.

The antitumor activity of 10 mg/kg oral OC powder formulation (OC-PF) administered daily for 11 days was assessed in orthotopic athymic nude mice bearing a CWR-R1ca-Luc tumor cells xenograft. OC-PF-treated mice showed a mean tumor volume of 119.7 ± 77.89 mm^3^ versus 713.6 ± 211.5 mm^3^ tumor volume in the placebo control-treated mice (Figure 3A). The mean tumor weight was 0.12 ± 0.09 g and 0.55 ± 0.18 g for OC-PF-treated and placebo control-treated mice, respectively (Figure 3B–D). The mice mean body weight in placebo control- and OC-PF-treated groups was not significantly different over the experiment course (Figure 3E). Histological investigation of the collected primary tumors showed moderately to poorly differentiated tumor cells, which exhibited a glandular pattern with areas of necrosis more abundant in the OC-PF-treated group (Figure 3F). Tumor cells exhibited hyperchromatic, pleomorphic nuclei with highly proliferative mitotic figures. It was possible to tentatively conclude that there was no significant variation between the OC-PF and placebo control-treated tumor histology.

### 3.10. Immunofluorsence Expression Comparison of ki67 and CD31 in OC Versus Placebo Control Treatments in Collected mCRPC Primary Tumors

An IHC study was used to evaluate the effects of OC-PF treatments on the expression level of ki67 as a tumor progression marker and the vascular endothelium marker CD31. Results showed the significant reduction of both ki67 and CD31 expression levels in OC-PF-treated primary tumor sections, unlike in the placebo control-treated tumors (Figure 3G,H).

### 3.11. OC Treatments Reduced the Expression of SMYD2 and Downstream Signals in Collected mCRPC Primary Tumors

**Figure 4:**
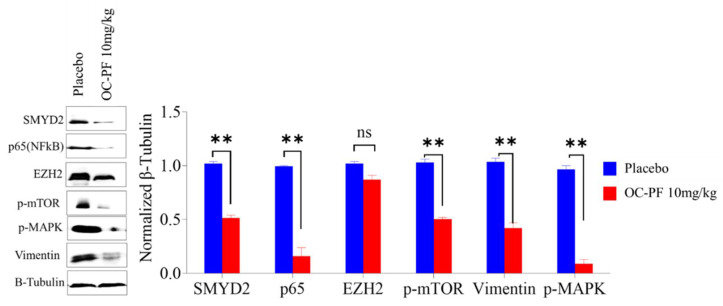
OC treatments reduced the expression levels of SMYD2, and downstream proteins in collected mouse primary tumors excision. Left panel: Western blot showing the expression of SMYD2, p65, EZH2, vimentin, p-mTOR, and p-MAPK. Right panel: Densitometric analysis of Western blots, quantifying the levels of SMYD2, p65, EZH2, the EMT marker vimentin, p-mTOR, and p-MAPK. Scanning densitometry obtained for all blots, carried out in triplicate, and the integrated optical density of each band was normalized with the corresponding density found for β-tubulin. Results shown in the bar graphs under their respective Western blot images. Vertical bars in the graph indicate the normalized integrated optical density of bands visualized in each lane. All raw immunoblots are included in Figure S8. ** *p* < 0.01 compared to their respective placebo control-treated mouse tumors. ns refers to non-statistical significance at *p* < 0.05 relative to control cells.

Western blotting analysis of collected primary tumor lysates confirmed the reduction of the SMYD2 expression level in OC-PF-treated primary tumors compared to in the placebo control-treated group (Figure 4). Collected tumors treated with OC-PF also showed suppressed levels of the SMYD2 protein substrates EZH2 and p65 and the EMT marker vimentin and lowered the activated mTOR and MAPK activities compared to the placebo control-treated group (Figure 4).

### 3.12. Oleocanthal PF Treatment Inhibited Locoregional Recurrence of the mCRPC CWR-R1ca Cells in Nude Mice after Primary Tumor Surgical Excision

**Figure 5:**
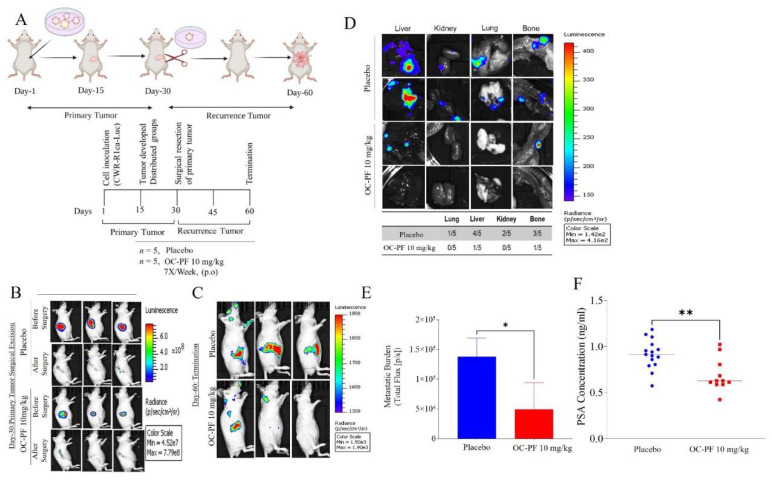
The mCRPC recurrence suppressive activity of OC-PF treatments in nude mice engrafted with CWR-R1ca-Luc cells and subjected to primary tumor surgical excision. (**A**) Experimental design layout. (**B**) Live animal bioluminescence comparison of OC-PF-treated versus the placebo control-treated mice before and after surgical excision. (**C**) Comparison of bioluminescence images of OC-PF-treated versus placebo control-treated representative mice at the study end, 60 days after first tumor xenografting or 30 days after primary tumor surgical excision. (**D**) Bioluminescence images of collected organs on the last study day comparing the distant recurrence suppressive effects of OC-PF versus the placebo control treatments. (**E**) Comparison of the distant recurrences (metastatic) burden for OC-PF versus placebo control-treated mice using quantitative bioluminescence imaging at the study end. (**F**) Comparison of the mice serum PSA levels for OC-PF versus the placebo control at the study end. * *p* < 0.05 and ** *p* < 0.01 for statistical significance compared to placebo control-treated group.

Mice were subjected to primary tumor surgical excisions (*n* = 10) in a previous experiment and used to test the ability of OC to prevent mCRPC recurrence. Mice continued to receive daily oral OC-PF treatments for an additional 30 days (Figure 5A). Since the parent tumor cells engrafted into the mice were luciferase-tagged, we used weekly luciferin-aided live animal imaging to confirm the efficiency of tumor surgical excision and to trace locoregional and distant recurrences [26]. IVIS bioluminescence live animal imaging of mice subjected to primary tumor surgical excision showed low luminescence, confirming the effective surgical removal of primary tumors (Figure 5B). All mice were healthy after surgery and, therefore, could be randomly parsed into two groups, *n* = 5 each. One group was treated with the placebo control and the other group treated with 10 mg/kg oral OC-PF by gavage, as detailed in the Methods. At the end of the study, four out of five mice treated with the placebo control developed distal tumor recurrence in multiple organs, including lung, kidney, liver, and bone. Meanwhile, only two out of five mice in the OC-PF-treated group developed distal recurrent tumors. One tumor developed in the liver and one in bone. More importantly, three out of five mice did not develop any sign of distant recurrence (metastasis, Figure 5C–E). All animals completed the whole experiment course until the study terminated 30 days after the excision surgery. The average body weights of the OC-PF-treated mice did not differ significantly from the placebo-treated mice (Figure 5F). Mice organs treated with placebo control and OC-PF showed no significant statistical difference (Table S1). OC-PF treatments significantly decreased the recurrence marker PSA level in mice serum, with an average of 1.64 ± 0.17 ng/mL in OC-PF-treated mice compared to 1.95 ± 0.13 ng/mL in the placebo control-treated group, representing a 15.9% reduction (Figure 5G).

### 3.13. In Vivo Safety of OC-PF Treatments

Daily OC-PF 10 mg/kg oral dosing produced no gross adverse and/or behavioral responses in male athymic nude mice over 41 dosing days. OC-PF-treated mice showed no significant change in the weight of various collected organs compared to the placebo control group (Table S1). The collected organs, including brain, heart, lung, liver, spleen, and kidney, were sectioned and stained to study any histopathological changes (Figure S4). No histopathological changes were observed in organs of either placebo control or OC-PF-treated mice (Figure S4).

## 4. Discussion

Despite the progress made towards androgen suppression, including LH/FSH modulators, anti-androgen receptor (AR) small molecules, CYP17A and CYP27A1 inhibitors, chemotherapies, and immunotherapy, these treatments lack absolute curative efficacy, especially in mCRPC patients, and have poor overall survival and high recurrence incidence [1,2,3,4]. Androgen deprivation therapy (ADT) is the standard of care for initial management of advanced or metastatic PC cases, but progression to CRPC occurs within 2–3 years of initiation of ADT. The microtubule depolymerization disruptor taxane docetaxel has historically been the first-line treatment for mCRPC, along with the androgen signaling antagonist enzalutamide and CYP17A1 inhibitors such as abiraterone acetate. Over a short period of therapeutic use, these agents fail to suppress mCRPC pathogenesis [31,49]. The National Comprehensive Cancer Network guidelines for PC control recommend combination platinum-based (carboplatin or cisplatin) chemotherapy as a first-line therapy for primary poorly differentiated small-cell or neuroendocrine PC (SCPC/NEPC) [31]. mCRPC is a heterogeneous phenotype with diverse progression and mechanisms of therapeutic resistance drivers [31]. Thus, it is imperative to target new pathways contributing to mCRPC recurrence and to develop novel interventions for the effective control of mCRPC progression and recurrence.

Cellular lysine methylation is driven by protein lysine methyltransferases (PKMTs), including the SET- and MYND-containing protein (SMYD) family. SMYD PKMTs are critical for gene regulation, chromatin remodeling, transcription, signal transduction, cell cycle control, and DNA damage response [17,18,19,20,21,50,51,52]. Disruption of SMYD1 perturbs cardiac morphogenesis and can induce embryonic lethality [53]. SMYD3 is associated with cancer cell proliferation and was reported to be overexpressed in hepatocellular, colorectal, prostate, and breast carcinomas [54]. SMYD4 is a tumor suppressor that plays a critical role in breast carcinogenesis, at least in part, through inhibiting the expression of PDGFRA [55]. SMYD5 is a negative regulator of inflammatory response genes [56]. SMYD5 is recruited to a subset of TLR4-responsive promoters via association with NCoR corepressor complexes, trimethylating histone H4K20 [56]. SMYD2 (KMT3C) methylates H3K36 and H3K4, in presence of HSP90, along with several histones, including H3, H4, and H2B and non-histone proteins such as EZH2 and p53, the retinoblastoma protein (RB), ERα, PARP1, MAPKAPK3, PTEN, and many others [15,16,17,18,19,20]. Lysine methylation by SMYD modifies the protein substrates’ bulkiness, hydrophobicity, and molecular recognition by methyllysine readers [21]. Methyllysine-containing proteins are recognized by downstream effectors possessing methyllysine reader domains, conferring their biological effects. The SMYD2 gene resides in 1q32–q41, a chromosomal region mostly amplified in esophageal and renal cell carcinoma, gastric, colon, pancreatic, lung, bladder, TNBC, and PC [10,11,12,13,14,15,16,17,18,19,20,21,22]. Targeting tumor SMYD2 is of high oncological therapeutic value [14,15,16,17,18,19,20,21]. Tumor cells heavily depend on SMYD2, unlike normal cells, justifying the SMYD2 inhibitors’ high therapeutic potential. A few small-molecule SMYD2 inhibitors that have been developed are LLY-507, AZ505, A-893, and BAY-598 [10,11,12,13,14,15,16,17,18,21]. They showed exceptionally high potency and selectivity in vitro but did not have a compelling effect on in vivo antitumor potency, and none of them earned FDA approval, justifying the continued need for the discovery of novel, SMYD2-modulating lead entities [19,20,47]. The SMYD family member SMYD3 proved a valid molecular target in PC since it modulates transcriptional and key signaling pathways and orchestrates multiple oncogenic inputs [20,22]. Dysregulated SMYD2 is linked with higher tumor recurrence rate [10,11,12,13,14,15,16,17,18,19,20].

This study analyzed the TCGA data and highlighted the dysregulation of the SMYD family in patient PC clinical samples, unlike in normal tissue samples (Figure S5). SMYD family dysregulation was notable in nodal prostate metastasis and patients with high Gleason score and shorter overall survival (Figure S6).

A Western blotting study indicated that SMYD2 is excessively amplified in the mCRPC cells CWR-R1ca, as well as in the AI PC-3, PC-3M, and DU-145 PC cells (Figure 1A). The Western blotting study also indicated the SMYD2 expression level in the mCRPC CWR-R1ca cells is 1.8-fold its level in the non-tumorigenic RWPE-1 prostatic epithelial cells. Thus, the mCRPC cells CWR-R1ca were selected for subsequent studies since they dysregulated SMYD2 and had a very high recurrence rate [33]. CWR-R1ca cells are aggressive, fibroblast-free, highly recurrent, metastatic CRPC cells with human origin [33]. CWR-R1ca cells are androgen-sensitive, express full-length AR and PSA, and represent the most aggressive CRPC phenotype with dysregulated SMYD2 pattern [33]. CRISPR/Cas9-aided KO of SMYD2 in CWR-R1ca cells (CWR-R1ca-KO) showed ~90% reduced proliferation, colony formation, migration, and invasion versus the wild-type CWR-R1ca cells, indicating the critical importance of SMYD2 for mCRPC viability and motility.

Epidemiological studies validated the Mediterranean diet’s ability to reduce the incidence of some tumor types [23,24,25,26,27,28,29,30,32,34]. Olive oil is a key fat source in the Mediterranean diet [23,24,25,26,27,28,29,30,32,34]. This study team previously reported the ability of OC to inhibit mCRPC PC-3 cell viability, migration, and invasion by competitive inhibition of c-MET receptor tyrosine kinase [3,26,32,34,48,57]. In this study, results acquired through collaboration with Eli Lilly OIDD indicated that OC inhibited the monomethylation of p53 Lys^370^ using scintillation proximity assay [47]. OC showed potent, dose-dependent, antiproliferative activities against the mCRPC/AI cell lines CWR-R1ca, PC-3, PC-3M, and DU-145. Interestingly, OC only adversely affected the non-tumorigenic RWPE-1 prostatic epithelial cells’ viability at nearly 10-fold its therapeutic concentrations. This clearly highlighted the high selectivity of OC to PC cells and reflected its high safety and therapeutic profile. OC showed potent, antimigratory, anti-invasive, and colony formation inhibitory activities in wound-healing, Matrigel invasion, and colony formation assays, respectively, against CWR-R1ca cells at subtoxic treatment doses. OC treatments in the range of 0.5–2 μM effectively reduced the SMYD2 expression in the mCRPC CWR-R1ca cells, as evidenced by Western blotting. Meanwhile, treatment of the mCRPC CWR-R1ca cells with the standard FDA anticancer drugs ENZ, DTX, and CPT at doses slightly higher than their IC_50_ values did not affect the SMYD2 expression level. This clearly proved the OC SMYD2 expression suppression in mCRPC is a selective pharmacological effect rather than a downstream effect due to cytotoxicity. Overall, these in vitro results indicate the prosurvival and pro-motility importance of SMYD2 in mCRPC. The results also validated the potential of OC to suppress the progression and motility of mCRPC, at least in part, via the downregulation of SMYD2 level.

Unlike the known, reported SMYD2 inhibitors, OC-PF showed impressive in vivo antitumor potency at a modest 10 mg/kg daily oral dosing regimen. Over 11 days of oral dosing, OC inhibited the progression of the mCRPC CWR-R1ca cells engrafted in nude mice by more than 83%. Immunofluorescence assessment of CWR-R1ca primary tumor sections proved OC-PF effectively suppressed the tumor cells’ proliferation marker ki67 and the endothelial vasculogenesis marker CD31 compared to the placebo control-treated group. These results confirmed the in vivo pharmacodynamics effects of OC-PF against mCRPC. This conclusion was further confirmed by Western blotting analysis of the primary tumors, which revealed a significant reduction of SMYD2 protein expression level. This was associated with decreased expression of the SMYD2 protein substrates EZH2, p65, p-mTOR, and p-MAPK in OC-PF-treated primary tumor samples compared to in the placebo control-treated animal tumors.

A comparison of OC anti-PC in vitro potency and standard anti-PC drugs indicated OC’s superior activity over enzalutamide and cisplatin (Figure 2A,B and Table 1). Despite the fact that the taxane drugs docetaxel and paclitaxel outscored OC in MTT, its in vitro antiproliferative potency, the high safety profile, lack of neuropathic side effects associated with the use of taxanes, and in vivo oral potency qualify OC as a viable mCRPC therapeutic option individually or in combination with standard PC therapies.

Invisible tumor stem cells and resistant, dying, or dormant tumor cells in microenvironment contact with host mouse tissue are likely the tumor repopulation origin [58,59]. A 30-day continued oral OC dosing regimen after surgical excision of the mCRPC primary tumor prevented 100% of locoregional recurrence in nude mice. OC treatments prevented the mCRPC CWR-R1ca distant recurrences in the lung and kidney compared to the placebo control-treated group. OC treatments significantly suppressed the distant recurrence in liver (1/5 versus 4/5 for placebo control). OC treatments also effectively suppressed the distant mCRPC recurrence at the bones (1/5 versus 3/5 for placebo control).

The main PC biochemical recurrence marker is the PSA serum levels. PSA level drops to zero in treated PC patients, while the detection of 0.2 ng/mL PSA level in a PC survivor is a relapse marker. OC-PF-treated animal sera showed significant reduction in the PSA level compared to in the mice treated with the placebo control, highlighting the potential of OC as a prospective, novel, small-molecule PC recurrence suppressor via modulating SMYD2 expression level.

## 5. Conclusions

This study reported, for the first time, the importance of SMYD2 for mCRPC pathogenesis. The study showed the EVOO phenolic OC as a novel, first-in-class lead for the control of mCRPC progression and recurrence. Unlike the previously reported SMYD2 small-molecule inhibitors, OC-PF showed powerful in vivo oral potency against mCRPC progression and recurrence in nude mouse xenograft models at a modest therapeutic dose level [25,29,30,32,34,35]. OC’s expected long-term safety profile is based on the EVOO historical human food consumption, high selectivity for malignant tumor cells, and low toxicity for non-tumorigenic prostatic epithelial cells at therapeutic doses [60]. A single-dose safety study in Swiss albino mice suggested a high safety profile for OC [60]. OC is a cost-effective lead with its ample and sustained plant supply and readily scalable purification methodology [32]. OC can potentially be developed as a nutraceutical for the use in mCRPC patients and survivors without the need for FDA approval. OC in vivo activity was associated with reducing the main PC biochemical recurrence marker PSA, validating its potential as an mCRPC recurrence suppressor. This study validated that the EVOO secoiridoids represented by OC are a novel lead scaffold class that can be optimized to create clinically useful and active in vivo SMYD2 inhibitors for application to prevent or suppress the mCRPC recurrence and extend the disease-free survival of PC survivors. OC is a novel SMYD2 modulatory nutraceutical appropriate for near-future clinical application to control mCRPC.

## Figures and Tables

**Table 1:** Comparison of OC in vitro antiproliferative activity against PC cell lines versus enzalutamide and standard chemotherapeutic drugs.

Cell Line	Oleocanthal		Paclitaxel	Docetaxel	Enzalutamide	Cisplatin
	IC_50_ µM		IC_50_ nM	IC_50_ nM	IC_50_ µM	IC_50_ µM
	(24 h)	(48 h)	(48 h)	(48 h)	(48 h)	(48 h)
CWR-R1ca	3.59	1.08	4.9	2.2	84.2	24.3
PC-3	0.64	0.58	6.6	2.0	70.1	6.1
PC-3M	1.03	0.80	3.7	2.8	90.1	50.8
DU-145	5.35	3.32	5.7	2.6	47.0	15.3

## Data Availability

Data used are cited in the article, references number [36,38,41,42].

## Supplementary Materials

The following supporting information can be downloaded at: https://www.mdpi.com/article/10.3390/cancers14143542/s1, Figure S1: Clinical contribution of SMYD family members to PC prognosis; Figure S2: The role of SMYD2 in PC pathogenesis; Figure S3: SMYD2 expression profile in various organ tumors and comparison of its expression in prostate normal tissues versus PC nodal metastasis; Figure S4: Toxicity evaluation of OC-PF treatments on different nude mouse organs; Figure S5: TCGA expression profile of SMYD family members in PC patients; Figure S6: Correlation of SMYD family members’ expression profile with Gleason score and PC progression-free survival; Figure S7: Raw Western blotting gels of β-tubulin and SMYD2 in different prostate cancer cells and RWPE-1 prostate epithelial cells with and without OC treatment; Figure S8: SMYD2 and downstream substrate proteins raw Western blotting gels. Table S1: Comparison of the effects of OC treatments versus the placebo control on various mice organ weights.
