# Supplementary material for: Oleocanthal Attenuates Metastatic Castration-Resistant Prostate Cancer Progression and Recurrence by Targeting SMYD2
Source: Cancers (Basel). 2022 Jul 21;14(14):3542. doi: 10.3390/cancers14143542 (PMC9317016; doi:10.3390/cancers14143542)
Supplement: Supplementary file 1 [file cancers-14-03542-s001.zip › cancers-1768978-supplementary.pdf]

Article Supplementary Materials

Oleocanthal Attenuates Metastatic Castration-Resistant Prostate Cancer Progression and Recurrence by Targeting SMYD2

Abu Bakar Siddique, Hassan Y. Ebrahim, Afsana Tajmim, Judy Ann King, Khaldoun S. Abdelwahed, Zakaria Y. Abd Elmageed and Khalid A. El Sayed

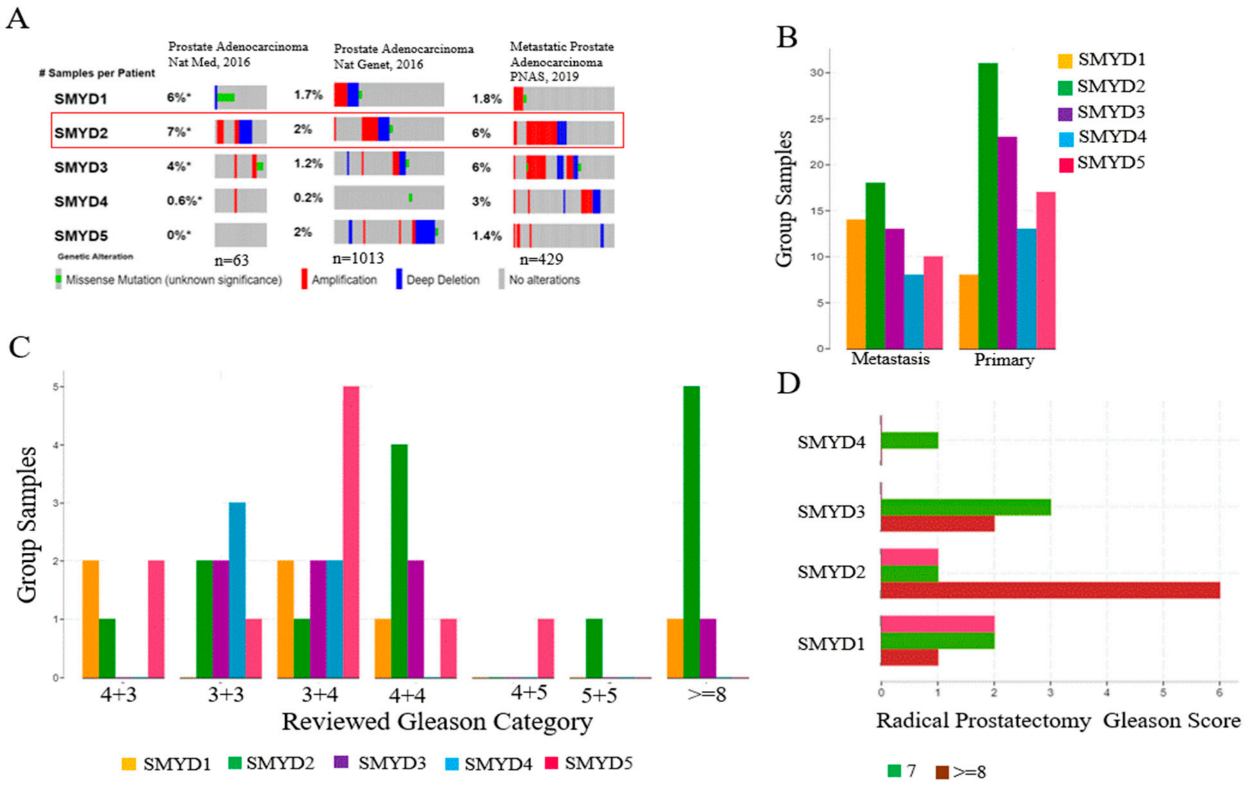

Figure S1. Clinical contribution of SMYD family members to PC prognosis. (A) SMYD family members association with alteration of genes by amplification, deep deletion, and mutation in PC. (B) SMYD family members expression profile in clinical patient primary and metatstatic prostate tumor samples. (C) Distribution of SMYD family members patient prostate tumor samples based on Gleason score. (D) Correlation of SMYD family members expression with patient samples radical prostatectomy Gleason scores.

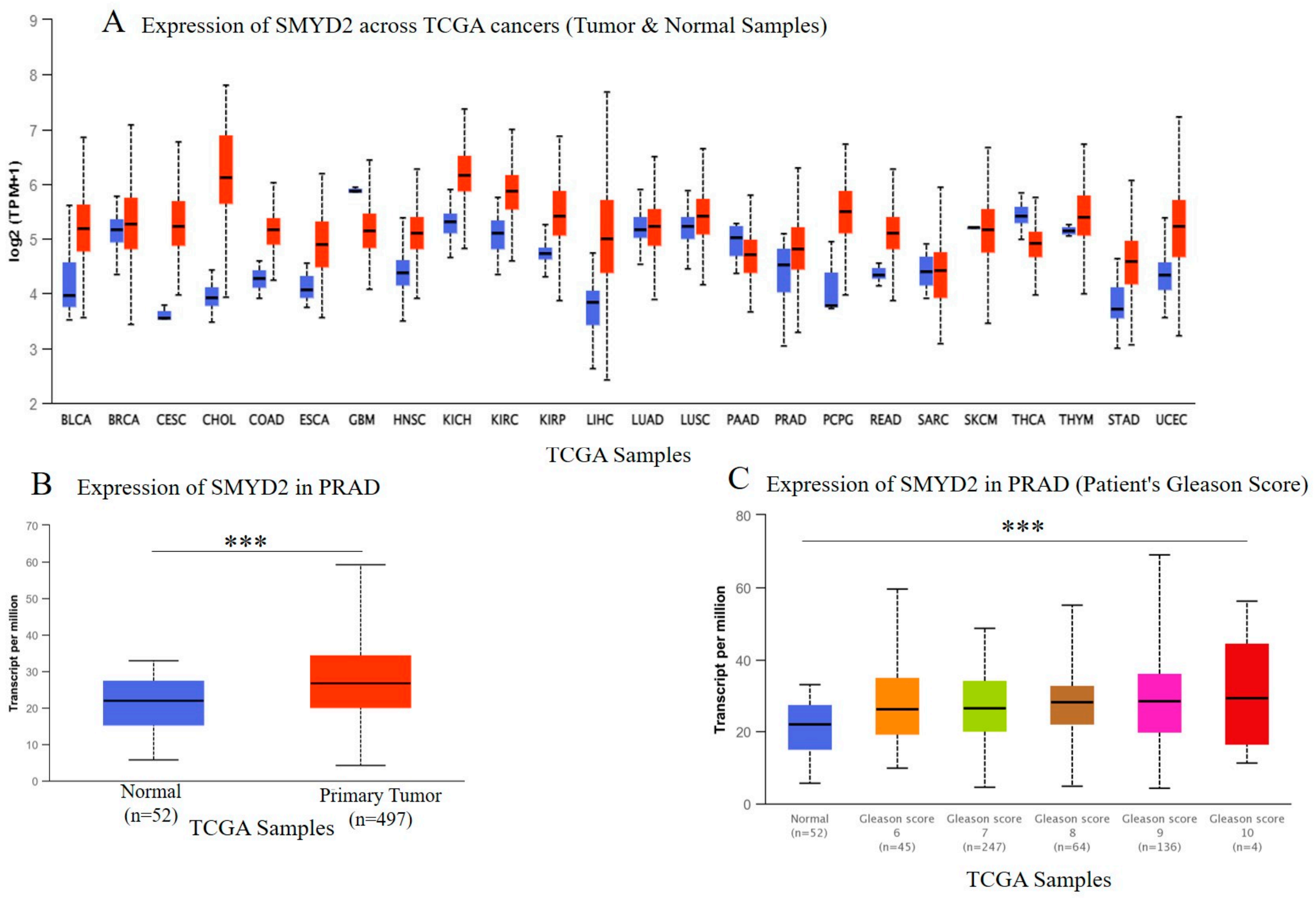

Figure S2. The role of SMYD2 in PC pathogenesis. (A) The SMYD2 mRNA expression in normal (blue) versus diverse types of cancer (red). (B) Comparison of SMYD2 mRNA expression in normal prostate tissues (n=52) versus primary PC (n=497). (C) Correlation between SMYD2 mRNA expression and PC patients Gleason's score. *** $p < 0.001$.

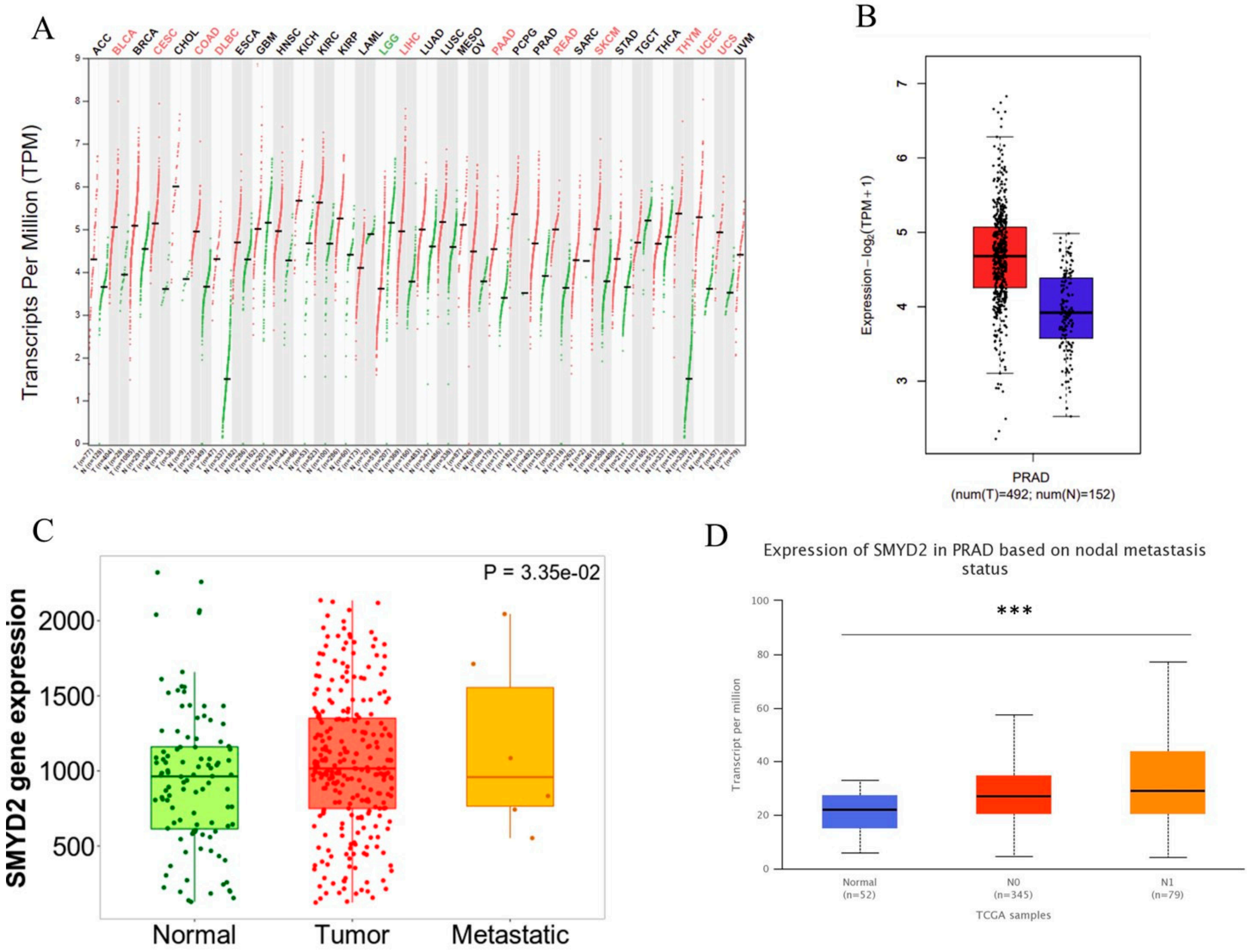

Figure S3. SMYD2 expression profile in various organ tumors and comparison of its expression in prostate normal tissues versus PC nodal metastasis. (A) Comparison of SMYD2 expression in different organ malignancies versus normal tissues. (B) Comparison of SMYD2 in normal prostate versus prostate adneocarcnoma. (C) Comparison of SMYD2 expression in normal tissues versus non-metatstic and metatstatic prostate adenocarcinoma. (D) SMYD2 expression proved highest in prostate nodal metastasis, followed by non-metastatic prostate adenocarcinoma and the lowest SMYD2 expression level was in normal prsotate tissue. *** $p < 0.001$.

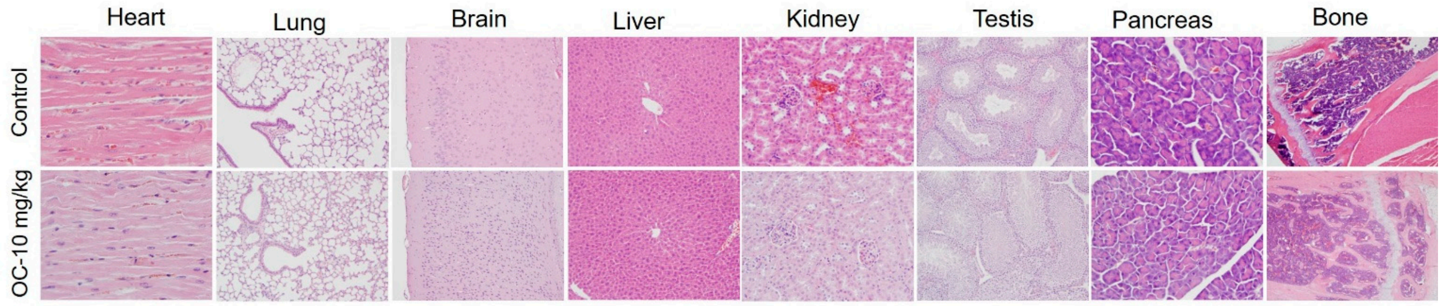

Figure S4. Toxicity evaluation of OC PF treatments on different nude mouse organs. Mice different organs including heart, lung, brain, liver, kidney, testis, pancreas and bone showed no histopathological variations beween OC PF and placebo control-treated groups.

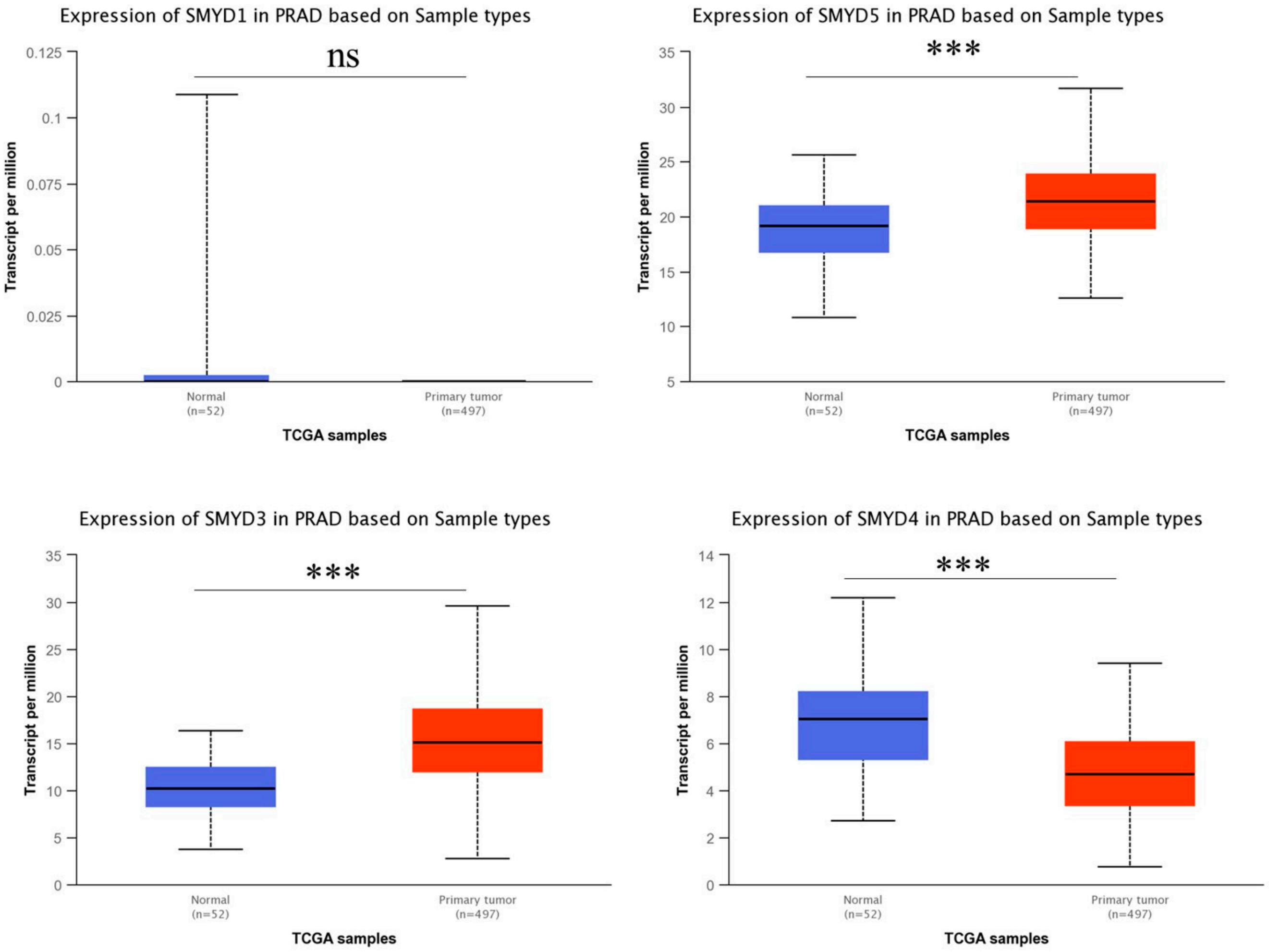

Figure S5. TCGA expression profile of SMYD family members in PC patients. *** $p < 0.001$, ns refers to non-statistical significance at $p < 0.05$ relative to control cells.

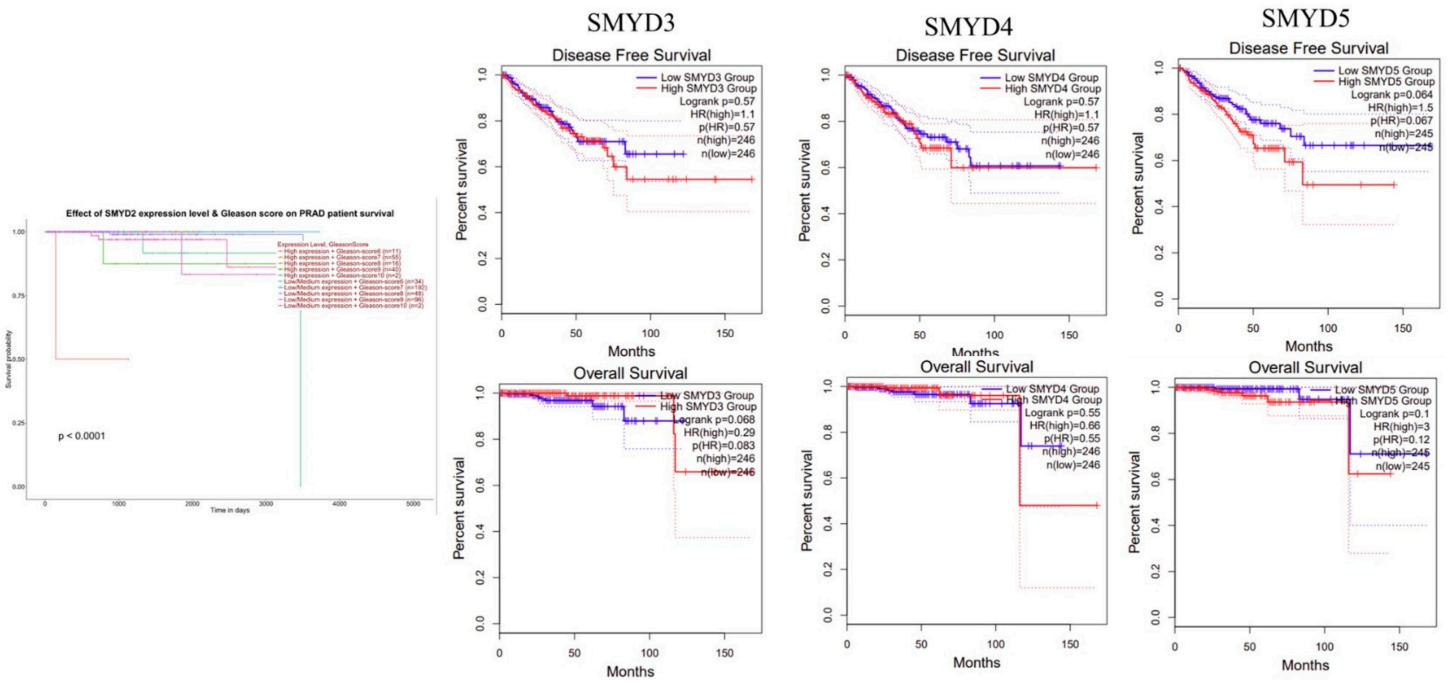

Figure S6. Correlation of SMYD family members expression profile with Gleason score and PC progression-free survival.

β-tubulin

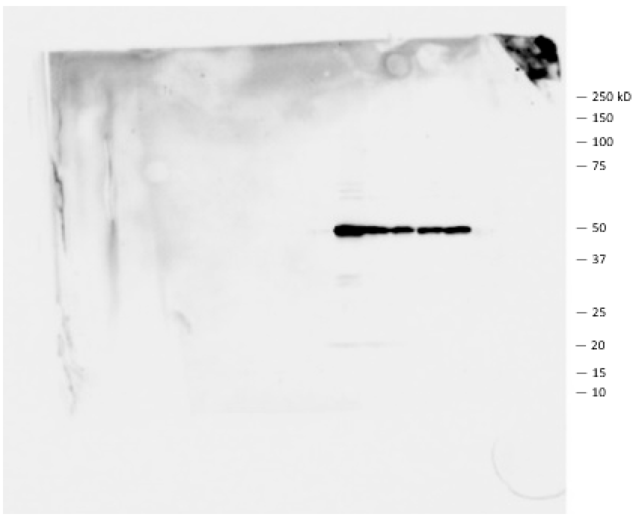

β-tubulin

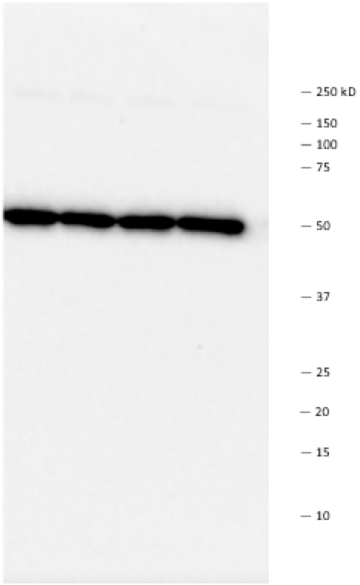

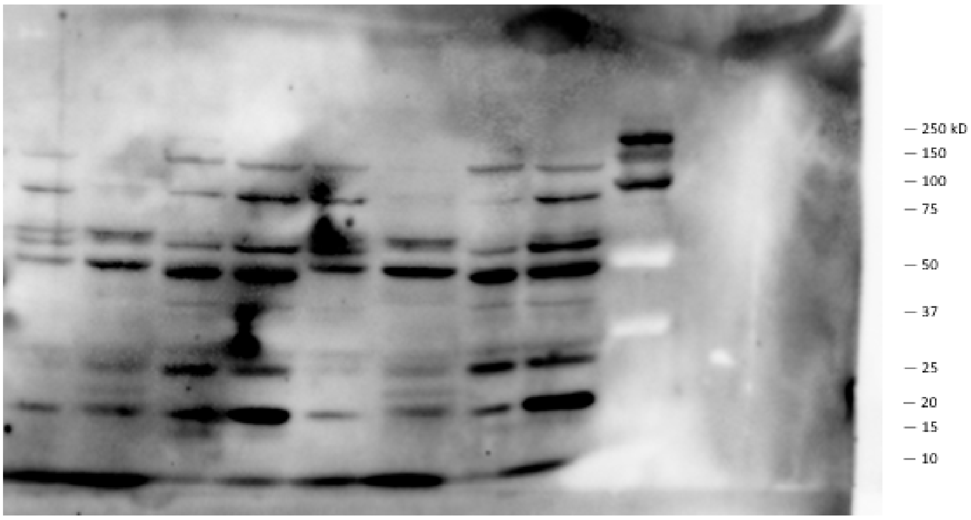

SMYD2

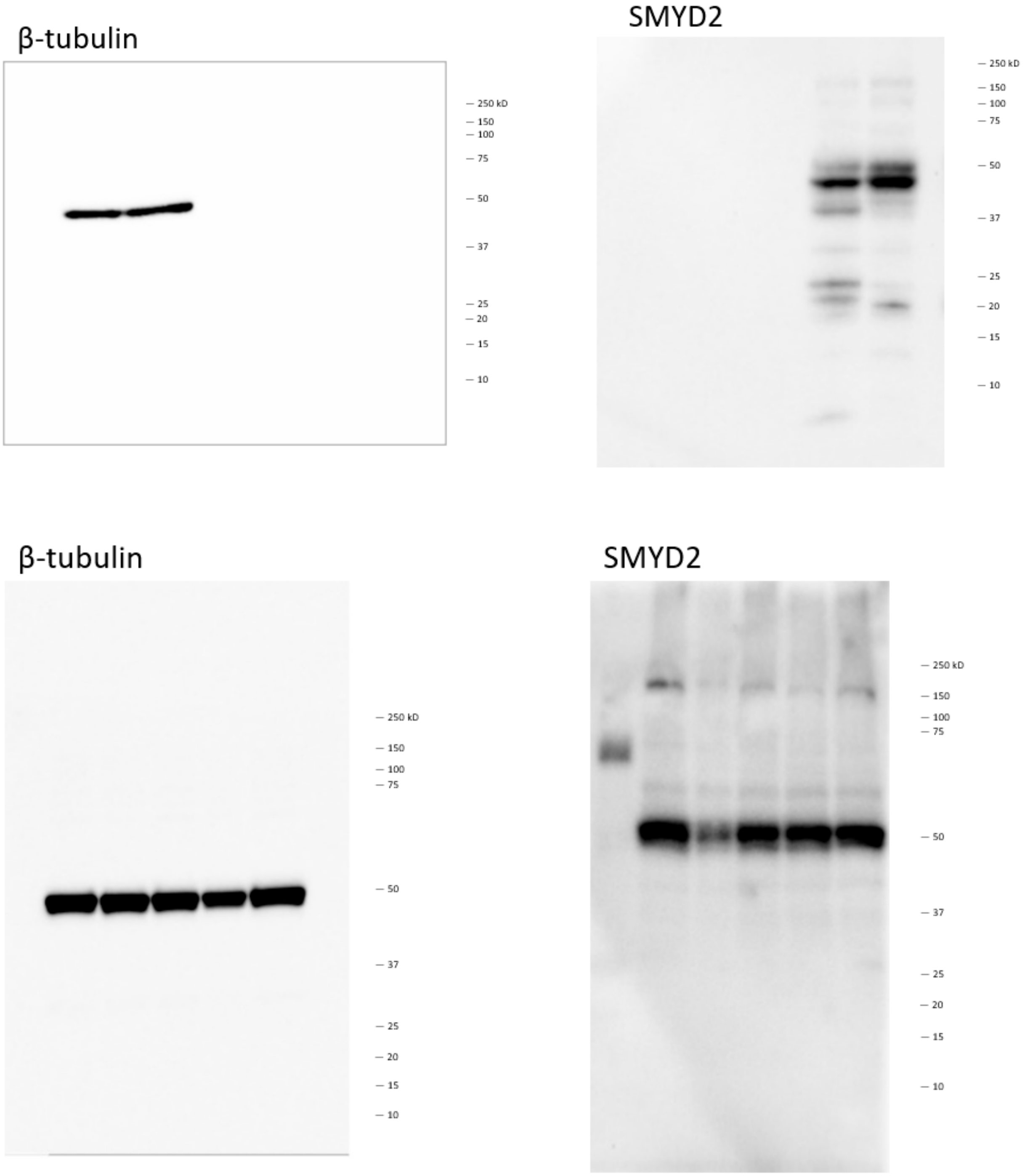

Figure S7. Raw Western blotting gels of β-tubulin and SMYD2 in different prostate cancer cells and RWPE-1 prostate epithelial cells with and without OC treatment.

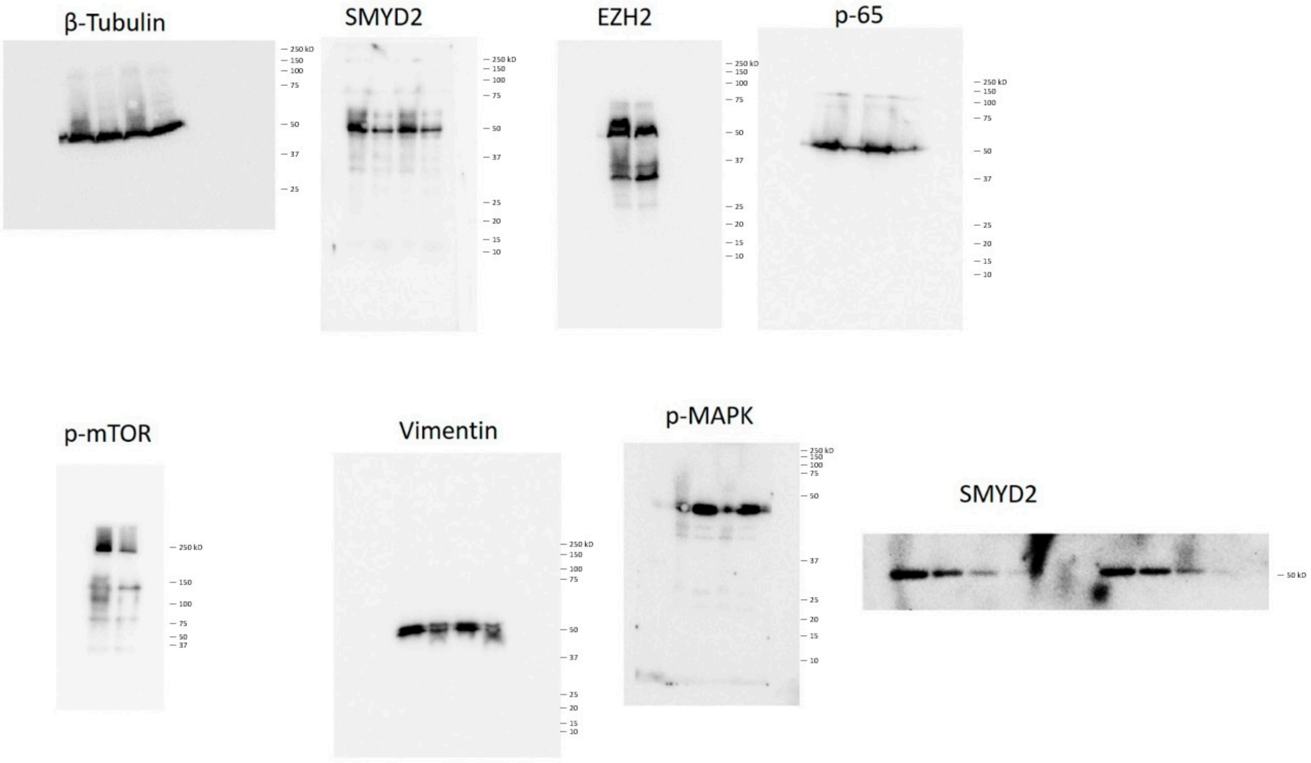

Figure S8. SMYD2 and downstream-substrate proteins raw Western blotting gels.

Table S1. Comparison of the effects of OC treatments versus placebo control on various mice organ weights.

Organs	Placebo Control	OC 10mg/kg
Brain (g)	0.36±0.06	0.35±0.05
Heart (g)	0.15±0.01	0.16±0.02
Lung (g)	0.29±0.06	0.24±0.04
Liver (g)	1.39±0.06	1.17±0.18
Kidney (g)	0.51±0.04	0.46±0.09
Pancreas (g)	0.11±0.01	0.09±0.03
Testis (g)	0.23±0.03	0.21±0.02
Prostate (g)	0.03±0.01	0.04±0.01
